# An immunochromatographic test using whole blood for rapid diagnosis of human paragonimiasis and its diagnostic usefulness

**DOI:** 10.1016/j.fawpar.2024.e00246

**Published:** 2024-09-23

**Authors:** Patcharaporn Boonroumkaew, Lakkhana Sadaow, Penchom Janwan, Rutchanee Rodpai, Oranuch Sanpool, Tongjit Thanchomnang, Hiroshi Yamasaki, Pewpan M. Intapan, Wanchai Maleewong

**Affiliations:** a Department of Parasitology, Faculty of Medicine, Khon Kaen University, Khon Kaen 40002, Thailand; b Mekong Health Science Research Institute, Khon Kaen University, Khon Kaen 40002, Thailand; c Department of Medical Technology, School of Allied Health Sciences, Walailak University, Nakhon Si Thammarat 80161, Thailand; d Department of Medical Technology, Faculty of Allied Health Sciences, Nakhonratchasima College, Nakhon Ratchasima 30000, Thailand; e Faculty of Medicine, Mahasarakham University, Maha Sarakham 44000, Thailand; f Department of Parasitology, National Institute of Infectious Diseases, Tokyo 162-8640, Japan

**Keywords:** Immunochromatographic test, Diagnostics, Paragonimiasis, *Paragonimus heterotremus*, *Paragonimus westermani*, *Paragonimus miyazakii*

## Abstract

Paragonimiasis is a harmful food-borne zoonosis caused by lung flukes of the genus *Paragonimus*. The disease is found on most continents, several million people are at risk of infection, and it is a re-emerging disease in developing countries. The gold standard for diagnosis of pulmonary paragonimiasis requires the finding of eggs in sputa and/or fecal samples. In ectopic paragonimiasis cases, eggs are typically not seen, and supportive information is required such as a history of eating freshwater crabs or crayfishes, radiographic findings and immunological tests. Here, we developed a proof of concept based on lateral flow assay, an immunochromatographic test kit, named the paragonimiasis whole-blood test kit, for detection of specific IgG antibody in simulated whole-blood samples (WBSs) using worm excretory-secretory antigens to diagnose human paragonimiasis. The laboratory diagnostic values of this kit were compared with the detected IgG in serum samples. In simulated WBSs, the diagnostic sensitivity and specificity were 97.8 % and 96.1 %, respectively, while for serum samples, these values were 100.0 % and 94.8 %, respectively. The comparative IgG antibody detections whether a result was positive or negative between simulated WBSs and serum samples did not differ significantly with a concordance of 97.8 % in laboratory conditions using a circumscribed set of samples. The tool is fast and easy to use. The next step involves observing and evaluating native whole blood samples and using specific recombinant antigens need to be evaluated for support diagnosis of paragonimiasis caused by *P. heterotremus, P. westermani* and *P. miyazakii* at the bedside or at local and remote hospitals with limited facilities. It will also be valuable for epidemiological surveys in Asia where paragonimiasis is endemic.

## Highlights

• A new assay for human paragonimiasis, a food-borne zoonosis caused by lung flukes.
• An immunochromatographic test using simulated whole blood and serum samples
• Detection of specific IgG antibody against worm excretory-secretory antigens
• Highly sensitive and specific test and possible application with fingerstick blood samples
• Supportive rapid diagnosis in endemic areas and field survey without sophisticated equipment

## 1 Introduction

Human paragonimiasis, the disease caused by the lung flukes in the genus *Paragonimus* (Trematoda: Paragonimidae), is a harmful food-borne zoonosis widely distributed in tropical, subtropical and some temperate zones. Various species of the genus *Paragonimus* have been reported from Asia, North and South America and Africa, but mainly occur in eastern Asia (Blair, 2022). Paragonimiasis is one of the main foodborne trematodiases targeted for control as part of the World Health Organization neglected tropical diseases road map for years 2021 to 2030 (Tidman et al., 2023). About 300 million people are at risk of infection and approximately 20 million people are infected with lung flukes, primarily in mainland China (Kong et al., 2015). In certain region of Africa (Southwest Cameroon), a prevalence of 14.9 % has been reported (Nkouawa et al., 2010). Lung flukes are now re-emerging to cause human disease (Blair, 2022) in some countries, including Thailand (Hanprom et al., 2023). The *Paragonimus westermani* complex (diploid and triploid worms) is the most common cause of human paragonimiasis in East and Southeast Asia. *Paragonimus skrjabini*/*Paragonimus skrjabini miyazakii* is the causative species in Japan, parts of China, northeast India and Thailand. *Paragonimus heterotremus* occurs in northeast India, southern China, Thailand, Vietnam and Lao PDR. *Paragonimus africanus* and *Paragonimus uterobilateralis* cause human disease in West Africa, *Paragonimus kellicotti* in North America, and *Paragonimus mexicanus* in Central and South America (Yoshida et al., 2019; Blair, 2022; Dubey, 2022).

Cases of paragonimiasis are most often found in areas where people have habitually consumed uncooked/undercooked freshwater crabs or crayfishes (the second intermediate hosts) harboring infective metacercariae. Eating raw and undercooked meat of wild boars or rodents (paratenic hosts containing infective juvenile worms) can also cause the disease.

Lung flukes cause paragonimiasis with a subacute to chronic lung inflammatory disease. The clinical cases mostly present with hypereosinophilia, chronic cough, chest pain, dyspnea and/or hemoptysis, and pleural effusion, the symptoms often resembling those of lung cancer and pulmonary tuberculosis, with which paragonimiasis is often confused (Blair, 2014; Kong et al., 2015). Ectopic cases can be found in which worms abnormally move to organs other than the lungs, frequently the brain (Blair et al., 2007).

Diagnosis depends on clinical signs and symptoms as well as a history of eating risky raw foods, chest X-ray and persistent eosinophilia (Kong et al., 2015). Proven diagnosis of paragonimiasis relies on the finding of *Paragonimus* eggs in feces or sputa, or both (Blair et al., 2007). However, sputum samples are sometimes hard to process and do not always contain eggs. *Paragonimus* eggs in feces fluctuate in numbers and may be misidentified as those of other intestinal flukes by inexperienced microscopists. Eggs cannot be found in feces and sputa in ectopic paragonimiasis cases.

Given the problems associated with parasitological diagnosis, serological tests are important to support clinical diagnosis. Various serological tests have been reported for supportive diagnosis such as enzyme-linked immunosorbent assay (ELISA) (e.g. Zhang et al., 1993; Ikeda, 1998; Intapan et al., 2013) and immunoblotting (e.g. Indrawati et al., 1991; Maleewong et al., 1992; Kong et al., 1998; Yun et al., 2000). However, these tests are time-consuming, require sophisticated equipment and can be done only in some laboratories. Recently, a point-of-care-testing (POCT) tool based on lateral flow assay, an immunochromatographic test (ICT) approach was reported for detection of anti-*Paragonimus* IgG antibody in serum samples for serodiagnosis of human paragonimiasis caused by *P. heterotremus*, *P. westermani* and *P. miyazakii* (Sadaow et al., 2020a).

However, the above-mentioned assays need serum samples and cannot be applied with whole-blood samples (WBSs), including fingerstick blood samples. In this study, we describe a proof of concept ICT kit, named the paragonimiasis whole-blood test (PB-ICT) kit, for detection of specific IgG antibody in WBSs using worm excretory-secretory (ES) antigens to diagnose human paragonimiasis. We compare the laboratory diagnostic values of this kit with those of the ICT kit that uses serum samples.

## 2 Materials and methods

### 2.1 Parasite collection and preparation of ES antigen

Adult *P. heterotremus* ES antigens were prepared by in-vitro culture as previously described (Sadaow et al., 2020a). The ES antigen solution, containing the protease inhibitors N-tosyl-*L*-phenylalanine chloromethyl ketone (0.1 mM) and phenylmethylsulfonyl fluoride (0.1 mM), was aliquoted and stored at −80 °C until used. The ES protein concentration was determined by the standard method (Lowry et al., 1951). Animal experimentation was approved by the Animal Ethics Committee of the Khon Kaen University (AEMDKKU 002/2018).

### 2.2 Clinical samples

The Ethics Committee for Human Research at Khon Kaen University (HE664044) and the Medical Ethics Committee of the National Institute of Infectious Diseases, Tokyo, Japan (No. 177), have approved the use of human sera. The criteria of STARD 2015 (Cohen et al., 2016) guided the conduct of this experiment. In this study, leftover serum samples from the frozen biobank at the Department of Parasitology, Faculty of Medicine and Mekong Health Science Research Institute Biobank project, Khon Kaen University, and National Institute of Infectious Diseases (NIID), Tokyo, Japan, kept at −70 °C were used.

The 321 serum samples were divided into three groups and manipulated to simulate WBSs. Group I (*n* = 90) consisted of serum samples from patients infected with *P. heterotremus* (*n* = 50), *P. westermani* (*n* = 20), and *P. miyazakii* (*n* = 20). Group II (*n* = 60) consisted of Thai (*n* = 30) and Japanese (*n* = 30) healthy controls who were free from any intestinal protozoan or helminth infection by stool examination (Elkins et al., 1986) at the time of blood collection. Group III (*n* = 171) consisted of sera from cases with other infections. Table 1 outlines the procedure.

**Table 1:** Evaluation of the immunochromatographic test tool using simulated whole-blood and serum samples.

Type of Samples examined	Diagnostic Methods (Reference)	No. Positive/Total Number	
		Simulated WBSs^a^ (% Positive)	Serum (% Positive)
Group I			
Paragonimiasis heterotremus	Western blot and the presence of eggs in sputa or feces (Wongkham et al., 1994)	50/50 (100)	50/50 (100)
Paragonimiasis westermani	Clinical signs and serology (Yokogawa et al., 1974)	19/20 (95)	20/20 (100)
Paragonimiasis miyazakii	Clinical signs and serology (Yokogawa et al., 1974)	19/20 (95)	20/20 (100)

Group II			
Thai healthy controls	Qualitative formalin ethyl-acetate concentration (FECT) technique (Elkins et al., 1986)	0/30	0/30
Japanese healthy controls	FECT (Elkins et al., 1986)	0/30	0/30

Group III			
Pulmonary tuberculosis	Sputum staining of acid-fast bacilli and bacterial culture	1/21 (4.8)	1/21 (4.8)
Opisthorchiasis viverrini	FECT (Elkins et al., 1986)	2/10 (20)	2/10 (20)
Taeniasis saginata	FECT (Elkins et al., 1986)	0/10	0/10
Sparganosis	Histopathology and PCR (Boonyasiri et al., 2014)	0/10	0/10
Cysticercosis	Clinical signs, imaging findings, serology histopathology and/or DNA analysis (Sadaow et al., 2023)	1/20 (5)	1/20 (5)
Ascariasis	FECT (Elkins et al., 1986)	0/10	0/10
Trichuriasis	FECT (Elkins et al., 1986)	0/10	0/10
Strongyloidiasis	FECT (Elkins et al., 1986) and/or the agar-plate culture method (Koga et al., 1991)	0/10	0/10
Hookworm infections	FECT (Elkins et al., 1986)	0/10	0/10
Capillariasis	FECT (Elkins et al., 1986)	0/10	0/10
Trichinellosis	Serology (Morakote et al., 1992)	2/10 (20)	3/10 (30)
Gnathostomiasis	Serology with clinical manifestations and history of dietary preferences (Janwan et al., 2016)	0/10	0/10
Angiostrongyliasis	Eosinophilic meningitis, clinical signs, and serology (Somboonpatarakun et al., 2020)	1/10 (10)	1/10 (10)
Schistosomiasis japonica	The presence of eggs in feces and serology (Rodpai et al., 2021)	0/10	1/10 (10)
Fascioliasis	Serologically positive fascioliasis (Sadaow et al., 2020b)	2/10 (20)	3/10 (30)
		Diagnostic values [95 % CI^b^]	
Sensitivity (%)		97.8 [92.2–99.7]	100.0 [96.0–100.0]
Specificity (%)		96.1 [92.7–98.2]	94.8 [91.1–97.3]
Positive predictive value (%)		90.7 [83.1–95.7]	88.2 [80.4–93.8]
Negative predictive value (%)		99.1 [96.8–99.9]	100.0 [98.3–100.0]
Positive likelihood ratios		25.10 [13.20–47.70]	19.30 [11.10–33.40]
Negative likelihood ratios		0.02 [0.01–0.09]	0.00
ROC area^c^		0.969 [0.950–0.989]	0.974 [0.960–0.988]

a WBSs, whole-blood samples.

b CI, confidence interval.

c ROC area, Receiver Operating Characteristic area.

Simulated blood was prepared with red blood cells (RBCs) of type O that were used to spike serum samples, as previously reported (Janwan et al., 2021). In short, a 500 μL sample of whole blood of type O was centrifuged at 13,200 ×*g* for 10 min at 4 °C, resulting in the removal of the plasma. The packed RBCs were rinsed using phosphate-buffered saline (PBS, pH 7.4) and centrifuged three times at 13,200 ×*g* for 10 min at 4 °C each time. The packed RBCs were re-suspended with PBS, and the suspension was aliquoted into 10 μL per tube, which were then centrifuged at 13,200 ×*g* for 10 min at 4 °C, the supernatant (6.5 μL) discarded, leaving 3.5 μL of packed RBCs, which were used for the preparation of simulated WBSs. The simulated WBSs were prepared by adding 6.5 μL of each serum sample to 3.5 μL of packed RBCs to simulate the normal levels of human blood components.

### 2.3 The paragonimiasis whole-blood test

**Fig. 1:**
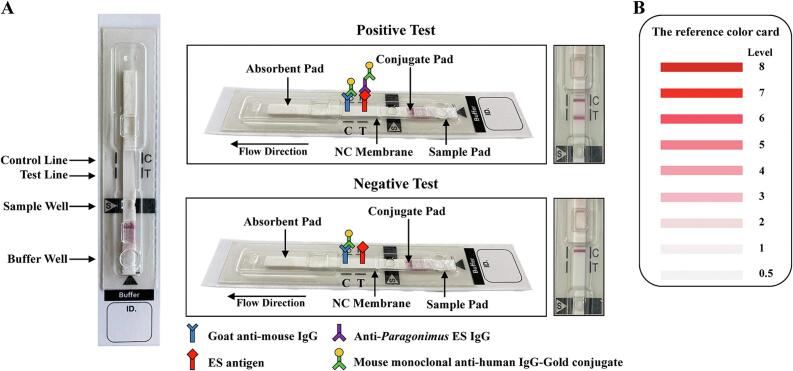
Detection of human paragonimiasis by the paragonimiasis whole-blood ICT kit. The components of the kit and how it works (A). The presence of both a colored test (T) line and a control (C) line indicate a positive result. If a colored band appeared only in the C line of the device, the result is negative. The result is read within 15 min by the naked eye and compared with the reference color card (B). ES antigen: adult *P. heterotremus* excretory-secretory antigens; NC membrane: nitrocellulose membrane.

We developed the PB-ICT diagnostic kit for human paragonimiasis, which detects specific IgG antibody against *P. heterotremus* ES antigens in either simulated WBSs or sera. The kit, as shown in Fig. 1, consisted of five components: nitrocellulose (NC) membrane (on the NC membrane were dispensed 1.5 mg/mL of *P. heterotremus* ES antigen as the test (T) line and 1.0 mg/mL of goat anti-mouse IgG (Lampire Biological Laboratories, Pipersville, PA) as the control (C) line) using a XYZ3210 Dispenser (Bio-Dot, Irvine, CA)), conjugate pad (the colloidal gold-conjugated mouse monoclonal anti-human IgG was sprayed onto a glass microfiber filter (Whatman Schleicher & Schuell, Dassel, Germany) to form the conjugate pad), sample pad, absorption pad, and backing card. All components were laminated, cut into strips 4 mm wide using a guillotine cutter (CM5000 Guillotine; Bio-Dot), and each strip was covered with a plastic housing (Adtec Inc., Oita, Tokyo, Japan). The PB-ICT kit was stored in a desiccated sealed aluminum foil-package at room temperature until used. The reproducibility and consistency of the kit was stable for 24 months at ambient temperature (4–30 °C) (data not shown). The complete kit consisted of an immunochromatographic strip and chromatography buffer.

Each simulated WBS or serum sample was diluted (1:50) with buffer. Five microliter of diluted simulated WBS or serum sample was added into the sample well, and 60 μL of chromatography buffer was added into the buffer well, and results were recorded 15 min later. The within-day and between-day precision of the PB ICT kit was determined via analysis of pooled positive and negative reference samples. Pooled positive and negative control sera were prepared by mixing equal volumes of sera from 10 paragonimiasis patients and 10 healthy volunteers, respectively.

Results read by the naked eye with reference to a color card, were compared between simulated WBSs and the corresponding to serum samples. A test line of any intensity level of color at or above level 1 was considered positive. The test was regarded as negative if only the control line was visible.

### 2.4 Diagnostic performance

The diagnostic values were calculated as previously described (Galen, 1980), and Stata Statistical Software: Release 10 (StataCrop LP, Lakeway Drive College Station, TX) was used to perform the sensitivity, specificity, positive and negative predictive values, positive and negative likelihood ratios, Receiver Operating Characteristic areas, and Kappa value of this study.

## 3 Results

**Fig. 2:**
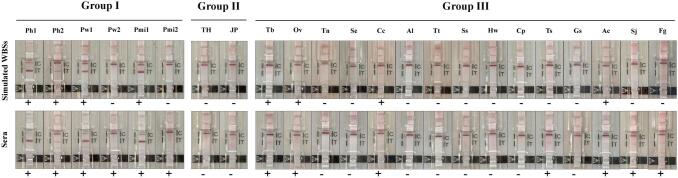
Results of paragonimiasis blood ICT kit tested using simulated whole-blood samples compared with the corresponding serum samples. Ph1-Ph2: paragonimiasis heterotremus cases; Pw1-Pw2: paragonimiasis westermani cases; Pmi1-Pmi2: paragonimiasis miyazakii cases; TH: Thai healthy individual; JP: Japanese healthy individual; Tb: pulmonary tuberculosis; Ov: opisthorchiasis viverrini; Tn: taeniasis saginata; Se: sparganosis; Cc: cysticercosis; Al: ascariasis; Tt: trichuriasis; Ss: strongyloidiasis; Hw: hookworm infections; Cp: capillariasis; Ts: trichinellosis; Gs: gnathostomiasis; Ac: angiostrongyliasis, Sj: schistosomiasis japonica, and Fg: fascioliasis. + and - indicate positive and negative results, respectively. WBSs, whole-blood samples.

Eighty-eight out of 90 simulated WBSs from paragonimiasis patients were positive, and 50, 19, and 19 were from patients infected with *P. heterotremus*, *P. westermani* and *P. miyazakii*, respectively. The diagnostic sensitivity was 97.8 % [95 %CI 92.2–99.7]. None of the 60 healthy controls from Thailand and Japan gave false-positive results. However, cross-reactions (9 of 171) were observed in 1 out of 21 pulmonary tuberculosis cases, 2 out of 10 opisthorchiasis viverrini cases, 1 out of 20 cysticercosis cases, 2 out of 10 trichinellosis cases, 1 out of 10 angiostrongyliasis cases, and 2 of 10 fascioliasis cases. The diagnostic specificity was 96.1 % [95 %CI 92.7–98.2] (Table 1, Fig. 2).

Similarly, ninety serum samples from paragonimiasis patients were all positive. The diagnostic sensitivity was 100.0 % [95 %CI 96.0–100.0]. None of the 60 healthy controls from Thailand and Japan gave false-positive results. However, the PB-ICT kit was positive in 12 of 171 (7.0 %) of the cases with other infections, as follows: pulmonary tuberculosis (1/21), opisthorchiasis viverrini (2/10), cysticercosis (1/20), trichinellosis (3/10), angiostrongyliasis (1/10), schistosomiasis japonica (1/10) and fascioliasis (3/10). The diagnostic specificity was 94.8 % [95 %CI 91.1–97.3] (Table 1, Fig. 2). Receiver operating characteristic analysis identified the optimal cutoff for kit interpretation on simulated WBS (0.969) and serum (0.974) samples. The simulated WBS and serum samples did not differ significantly in this respect (Kappa value 0.9490, agreement level almost perfect), with a concordance of 97.8 % (314/321; see Table 2).

**Table 2:** Comparison between simulated whole-blood and serum samples determination by the paragonimiasis whole-blood test.

Type of samples	Serum samples	
Simulated whole-blood samples	Positive Number	Negative Number
Number of Positive	96	1
Number of Negative	6	218

The comparison between simulated whole-blood and serum samples did not differ significantly (Kappa value 0.9490, agreement level almost perfect), with a concordance of 97.8 %.

## 4 Discussion

In the present study, a proof of concept ICT for detection of anti-*Paragonimus* IgG antibody in simulated WBSs has been reported for rapid diagnosis of human paragonimiasis. Although this gave positive results for paragonimiasis with *P. heterotremus* and two other lung flukes species, *P. westermani* and *P. miyazakii*, it was difficult to differentially diagnose between these species. This novel ICT kit gave higher diagnostic values (97.8–100.0 % sensitivity and 94.8–96.1 % specificity) than those of previous ICT kit (97.9 % of sensitivity and 87.6 % of specificity) (Sadaow et al., 2020a). Differences in sensitivity and specificity of these tests might depend on optimum conditions, such as buffer system, concentrations of antigen, kinds of membrane and type of plastic cover and differences in the panels of used samples.

In the present kit, absence of cross reactions with healthy serum samples has led to the high diagnostic sensitivity and specificity of 100 % and 94.8 %, respectively. Simulated WBSs yielded corresponding values of 97.8 % and 96.1 %, respectively. These results imply that our ICT kit can be used for screening and diagnostic purposes not only in Thai and Japanese populations but also in other countries in Asia where *P. heterotremus*, *P. westermani*, and *P. miyazakii* are the causative agents of human paragonimiasis.

Few cross reactions were found with some samples from opisthorchiasis viverrini, cysticercosis, trichinellosis, angiostrongyliasis, schistosomiasis japonica, and fascioliasis cases (Table 1), but diagnostic confusions with these helminthic diseases does not arise, as these diseases normally present clinical symptoms and signs very different from those of paragonimiasis. One out of 21 Thai pulmonary tuberculosis cases from Thailand, an area endemic for paragonimiasis, showed a cross reaction, raising the possibility that a paragonimiasis case might have been misinterpreted as pulmonary tuberculosis in the past or that lung flukes may have co-infected this patient. Clinicians who work in endemic areas where pulmonary tuberculosis and paragonimiasis co-occur should carefully consider diagnostic results. Patients should be differentially diagnosed on the basis of clinical symptoms and signs and a history of eating raw hazardous foods in combination with radiological and other laboratory blood findings, including eosinophilia.

However, clinicians and laboratory technologists should be aware, in diagnosing paragonimiasis using this kit, that the test has so far only been evaluated in a laboratory setting using a defined set of sera and simulated blood samples. The performance of the test still needs to be evaluated in real blood samples. Sensitivity and specificity might vary according to the populations in which the test is evaluated. In the present study, *P. heterotremus* ES antigens were obtained from experimentally raised adult worms. To improve this tool, highly sensitive and specific lung fluke antigens produced by recombinant technology should be developed. This would ensure a stable supply of antigen and simplify quality control. This improvement would likely lead to increased diagnostic values. However, also, the cost effectiveness of the test in health outcomes needs to be determined.

In conclusion, our proof of concept method provides a rapid and simple tool. The test has high sensitivity and specificity and can be used to detect anti-*Paragonimus* IgG antibody in human serum and simulated whole-blood samples. The test can support clinical diagnosis of paragonimiasis at the bedside and can also be used as a surveillance tool in areas where the risk of paragonimiasis is high and for diagnosis in remote areas where diagnostic/medical facilities are not well established. Moreover, the developed tool possible be applied with fingerstick blood samples.

## CRediT authorship contribution statement

**Patcharaporn Boonroumkaew:** Writing – review & editing, Writing – original draft, Validation, Resources, Methodology, Investigation, Funding acquisition, Formal analysis, Data curation, Conceptualization. **Lakkhana Sadaow:** Writing – review & editing, Writing – original draft, Validation, Software, Resources, Methodology, Investigation, Formal analysis, Data curation, Conceptualization. **Penchom Janwan:** Writing – review & editing, Visualization, Validation, Software, Resources, Methodology, Investigation, Formal analysis, Data curation. **Rutchanee Rodpai:** Writing – review & editing, Visualization, Validation, Resources, Methodology, Investigation, Formal analysis, Data curation. **Oranuch Sanpool:** Writing – review & editing, Visualization, Validation, Resources, Methodology, Investigation, Formal analysis, Data curation. **Tongjit Thanchomnang:** Writing – review & editing, Visualization, Validation, Resources, Methodology, Investigation, Formal analysis, Data curation. **Hiroshi Yamasaki:** Writing – review & editing, Writing – original draft, Visualization, Validation, Resources, Methodology, Investigation, Formal analysis, Data curation. **Pewpan M. Intapan:** Writing – review & editing, Visualization, Validation, Software, Investigation, Formal analysis, Data curation, Conceptualization. **Wanchai Maleewong:** Writing – review & editing, Writing – original draft, Visualization, Validation, Supervision, Project administration, Funding acquisition, Formal analysis, Data curation, Conceptualization.

## Declaration of competing interest

The authors declare that they have no known competing financial interests or personal relationships that could have appeared to influence the work reported in this paper.
